# 505. Real-World Use of Cabotegravir Long-Acting for Pre-Exposure Prophylaxis: Data from Trio Health Cohort

**DOI:** 10.1093/ofid/ofae631.157

**Published:** 2025-01-29

**Authors:** Moti Ramgopal, Carolyn A Brown, Andrew Frick, Janna Radtchenko, Gayathri Sridhar, Leigh Ragone, Jean A van Wyk, Karam Mounzer, Paul Benson, Steven Santiago, Sebastian Ruhs, Gregory Huhn, Kenneth H Mayer, Rick A Elion, Vani Vannappagari

**Affiliations:** Midway Immunology and Research Center, Fort Pierce, FL; ViiV Healthcare, Atlanta, Georgia; Trio Health, La Mirada, California; Trio Health, La Mirada, California; ViiV Healthcare, Atlanta, Georgia; ViiV Healthcare, Atlanta, Georgia; ViiV Healthcare, Brentford, UK, Brentford, England, United Kingdom; Philadelphia Fight Community Health Centers, Philadelphia, Pennsylvania; Be Well Medical Center, Berkley, MI; CareResource, Miami, Florida; Chase Brexton Health Care, Baltimor, Maryland; The Ruth M. Rothstein CORE CENTER, Chicago, Illinois; Harvard Medical School/Fenway Research Institute, Boston, MA; Trio Health, La Mirada, California; ViiV Healthcare, Atlanta, Georgia

## Abstract

**Background:**

Cabotegravir (CAB) long acting (LA) for pre-exposure prophylaxis (PrEP) was approved in the US in Dec 2021 to reduce the risk of sexually acquired HIV-1 infection. The Centers for Disease Control and Prevention (CDC) guidelines state that both HIV antigen (Ag)/antibody (Ab) and HIV RNA testing should be conducted prior to every injection. This analysis presents data on testing practices, effectiveness, adherence, and persistence in real world setting in the US.

**Table 1:**
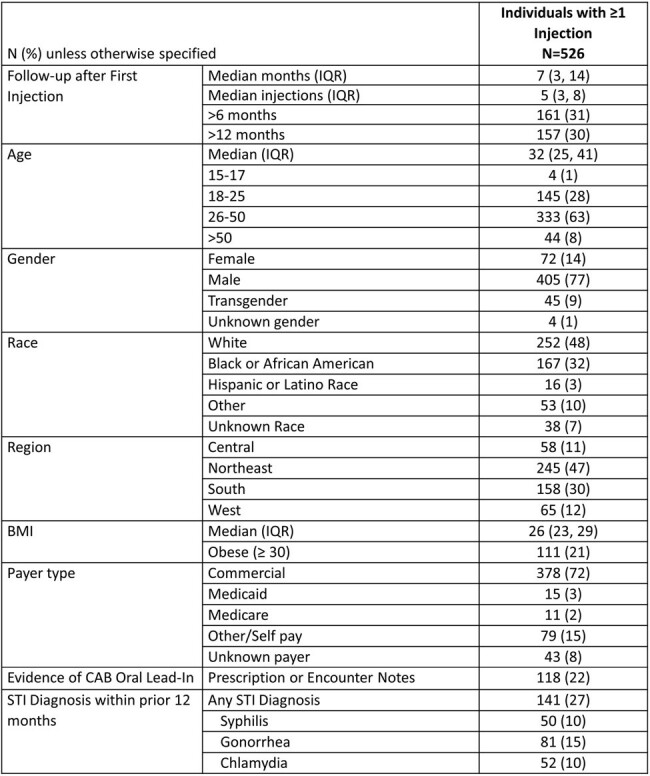
Baseline Characteristics for those with at least 1 injection of CAB LA PrEP

**Methods:**

Individuals initiating CAB LA PrEP were identified from electronic health records in the Trio Health cohort between Dec 2021- Jan2024. HIV testing within ±7 days of injection and HIV incidence were assessed among individuals with ≥ 1 injection of CAB LA. Incident HIV was defined as detectable HIV RNA +/- positive HIV Ag/Ab test. Adherence was assessed among those with ≥ 2 injections of CAB LA. As per label, the first 2 initiation injections are to be administered monthly followed by continuation injections every 2 months. On-time injections were defined as occurring within ±7 days of target date and discontinuations as 127 days without injections.

**Table 2:**
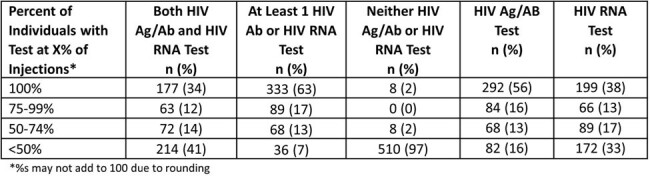
HIV Testing during Follow-up among Individuals with ≥1 injection of CAB LA for PrEP (N=526)

**Results:**

Among the 526 individuals with ≥ 1 injections (median 5 [IQR: 3-8]; median follow-up 7 months [IQR: 3-14] [Table 1]), 34% had both HIV RNA and HIV Ag/Ab tests performed, and 63% had either test prior to all injections [Table 2]. HIV RNA tests were used less frequently than HIV Ag/Ab tests. Of 474 with ≥ 2 CAB LA injections, 76 (16%) had a delayed 2nd injection with a median of 22 days delay [IQR: 9-33] [Table 3]. Of 396 with continuation injections, 131 (33%) had delays with median of 1 delayed injection and median delay of 12 days [IQR: 3-29]. Ten (3%) individuals had 1 missed injection and none missed ≥ 2 injections. No incident HIV diagnoses were identified. The majority were persistent at 6 months (92%) and at 12 months (85%).

**Table 3:**
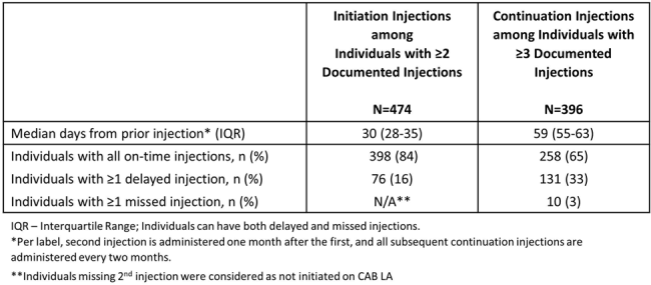
Delayed Injections among Individuals with ≥2 Injections of CAB LA for PrEP

**Conclusion:**

These real-world data with long duration of follow-up and a large sample size further support that CAB LA for PrEP remains effective at preventing HIV acquisition. Injections were administered on time among the majority of initiators. HIV testing practices in this real-world setting did not align with the CDC testing guidelines among a significant proportion of users. No incident HIV diagnoses were identified, regardless of testing approach or injection timing.

**Disclosures:**

**Moti Ramgopal, MD, FACP, FIDSA**, Gilead Sciences: Advisor/Consultant|Gilead Sciences: Grant/Research Support|Gilead Sciences: Honoraria|Janssen: Advisor/Consultant|Janssen: Grant/Research Support|Janssen: Honoraria|Merck: Advisor/Consultant|Merck: Grant/Research Support|ViiV Healthcare: Advisor/Consultant|ViiV Healthcare: Grant/Research Support|ViiV Healthcare: Honoraria **Carolyn A. Brown, MSPH, PhD**, ViiV Healthcare/GSK: Stocks/Bonds (Public Company) **Andrew Frick, MS**, Trio Health: Employee **Janna Radtchenko, MBA**, Trio Health: Employee **Gayathri Sridhar, MBBS, MPH, PhD**, GlaxoSmithKline: Stocks/Bonds (Public Company)|ViiV Healthcare: Full Time Employee **Leigh Ragone, MS**, GlaxoSmithKline: Stocks/Bonds (Public Company)|ViiV Healthcare: Full time employee **Jean A. van Wyk, MBChB, MFPM**, ViiV Healthcare: Employee|ViiV Healthcare: Stocks/Bonds (Public Company) **Karam Mounzer, MD**, Epividian: Board Member|Epividian: Honoraria|GS: Advisor/Consultant|GS: Grant/Research Support|GS: Honoraria|Merck: Advisor/Consultant|THERA Technologies: Grant/Research Support|ViiV healthcare: Advisor/Consultant|ViiV healthcare: Grant/Research Support|ViiV healthcare: Honoraria **Paul Benson, DO**, ViiV Healthcare: Advisor/Consultant|ViiV Healthcare: Grant/Research Support|ViiV Healthcare: Honoraria **Steven Santiago, MD**, Gilead Sciences: Advisor/Consultant|Gilead Sciences: Honoraria|Janssen: Honoraria **Gregory Huhn, MD, MPHTM**, Eli Lilly: Grant/Research Support|Gilead Sciences: Advisor/Consultant|Gilead Sciences: Grant/Research Support|Gilead Sciences: Honoraria|Janssen: Advisor/Consultant|Janssen: Grant/Research Support|Janssen: Honoraria|Merck: Advisor/Consultant|Merck: Honoraria|ViiV Healthcare: Grant/Research Support **Kenneth H. Mayer, MD, MPH**, Gilead Sciences: Advisor/Consultant|Gilead Sciences: Grant/Research Support|Gilead Sciences: Honoraria|Merck: Advisor/Consultant|Merck: Grant/Research Support|Merck: Honoraria|ViiV Healthcare: Grant/Research Support **Rick A. Elion, MD**, Gilead Sciences: Advisor/Consultant|Gilead Sciences: Grant/Research Support|Gilead Sciences: Honoraria|Janssen: Honoraria|Proteus: Grant/Research Support|Trio Health: Employee|ViiV Healthcare: Advisor/Consultant **Vani Vannappagari, MBBS, MPH, PhD**, GSK: Stocks/Bonds (Public Company)|ViiV Healthcare: Full time Employee|ViiV Healthcare: Stocks/Bonds (Public Company)

